# Comparison of micronutrients in adult enteral formulas widely used in clinical practice

**DOI:** 10.1002/fsn3.3545

**Published:** 2023-07-05

**Authors:** Hong Yang, Ling Hou, Hong Mei Sun, Shu Hong Ye

**Affiliations:** ^1^ Department of Food Science and Technology Dalian Polytechnic University Dalian China; ^2^ Xi'an Libang Clinical Nutrition Co., Ltd. Xi'an China

**Keywords:** daily energy dose, enteral formula, enteral nutrition support, micronutrients

## Abstract

In recent decades, great progress in the area of enteral nutrition has provided a large variety and commercial availability of enteral formulas, usually produced by the nutrition divisions of several pharmaceutical or dairy manufacturers, with specific compositions for each type of disease or patient condition. Despite the widespread use of enteral formulas, both in hospitals and at home, studies performed on the micronutrient compositions of adult enteral formulas are few in China. The content of micronutrients in 31 commercially available adult enteral formulas in the Chinese market was compared with the Chinese dietary reference intakes (DRIs), the tolerable upper limits (UL), the limit requirements in Food Safety National Standards General Rules of Foods for Special Medical Purposes (GB 29922‐2013), and the European Society for Clinical Nutrition and Metabolism (ESPEN) micronutrient guideline (2022). The micronutrient content was calculated by multiplying the value provided on the nutrition label for each product by the daily energy dose of 1500 and 1800 Kcal/day. The research results showed that most adult enteral formulas were generally suitable for patients on long‐term total enteral nutrition support in the Chinese market, and foods for special medical purpose (FSMP) formulas were more suitable than enteral nutrition preparation (ENP) formulas. However, the vitamin D, vitamin K, and iron content in these formulas should be appropriately increased to the limit recommended by the ESPEN micronutrient guideline. The results could provide a basis for manufacturers to research and develop more suitable enteral formulas and help clinical dietitians administer more effective enteral nutrition support for patients on long‐term total enteral nutrition in clinical practice, especially individualized treatment.

## Graphical abstract

The results could provide a basis for research and to develop more suitable enteral formulas by manufacturers and help to administer more effective enteral nutrition support for patients on long‐term total enteral nutrition by clinical dietitians in clinical practice, especially individualized treatment.

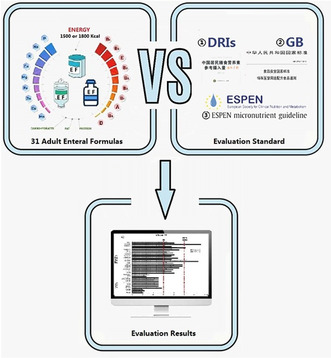

## 1 INTRODUCTION

Due to improved living standards and better nutrition in China, the obvious clinical symptoms caused by micronutrient deficiencies are becoming less common in the normal population, but someone with a disease, postoperative complications, or eating difficulties still often faces varying degrees of malnutrition. Enteral nutrition (EN) is the name given to the system that delivers nutrients directly into the gastrointestinal tract. Its purpose is to maintain or achieve a proper nutritional status in patients who have a functional gastrointestinal tract but are unable to meet their nutrient needs with a regular diet (Boullata et al., 2017; Cámara‐Martos & Iturbide‐Casas, 2019; Cederholm et al., 2017; Doley, 2022). The proposed benefits of the infusion of enteral nutrients include the prevention of adverse structural and functional alterations of the gut barrier induced by injury, increased epithelial proliferation, maintenance of mucosal integrity, decreased gut permeability, improved mesenteric blood flow, and improved local and systemic immune responsiveness (Chen & Peterson, 2009; Delompre et al., 2019; Hurt et al., 2019). At present, in the Chinese market, there are varieties of products used in enteral nutrition support that are classified as “enteral nutrition preparations (ENPs; drug approval)” or “foods for special medical purposes (FSMPs; special food approval).” For each kind of enteral formula, regardless of its approval and specific composition, when used as a single feeding method, enteral formulas need to be nutritionally complete and deliver the macro‐ and micronutrients that would be obtained through a regular diet in the required amounts. The patient's energy and protein needs, as well as the dietary reference intakes (DRIs) of micronutrients, are usually met with a daily intake of 1500–1800 Kcal/day, unless otherwise stated in the product information (National Health Commission of the People's Republic of China, 2013).

Micronutrients play a key role in human nutrition by regulating several metabolic processes (dos Santos Ribeiro, 2023). The effective metabolism of the major nutrients for protein and energy provision requires an adequate supply of all essential trace elements and vitamins. Since most patients requiring nutritional support present with a variably depleted micronutrient status, it is important to provide adequate amounts of all micronutrients from the start of nutrition support (Berger et al., 2022). However, there is still a significant gap in the research and analysis of micronutrient compositions in adult enteral formulas in China compared with other advanced countries, and studies are mainly limited to certain macronutrients. The micronutrient content in enteral formulas may be above or below the needs of patients with stable clinical conditions. In particular, the deviation from the DRIs should be given more attention for patients on long‐term enteral nutrition support, which may result in the clinical effects of deficiency or excess of micronutrients (Breik, Tatucu‐Babet, Paul, et al., 2022a; Breik, Tatucu‐Babet, & Ridley, 2022b; Casae & Bellomo, 2019). For this reason, an evaluation of the amount of vitamins, mineral elements, and trace elements in the currently available products widely used in clinical practice requires consideration.

With all the information above, this work's objective is to compare the content of micronutrients in 31 commercially enteral formulas widely used in clinical practice, which contain 12 registered ENPs' formulas and 19 registered FSMPs' formulas for patients, with the Chinese DRIs, the tolerable upper limits (UL), the limit requirements in Food Safety National Standards General Rules of Foods for Special Medical Purposes (GB 29922‐2013), and the European Society for Clinical Nutrition and Metabolism (ESPEN) micronutrient guideline (2022). Furthermore, analysis and suggestions are put forth to provide references for the formulas' research and development by manufacturers and for choosing and using the formulas by clinical workers.

## 2 MATERIALS AND METHODS

In this study, 31 commercially available enteral formulas (12 registered ENPs and 19 registered FSMPs) were nutritionally complete and/or recommended for different disease patients over 10 years old, including 17 liquid formulas (validity of 12 months) and 14 powder formulas reconstituted (validity of 24 months), which were manufactured by 19 different companies. The data come from the labels of 31 enteral formulas published on the official website of the State Administration for Market Regulation and the National Medical Products Administration. The deadline for data collection was December 31, 2022. According to the DRIs, UL, GB 29922‐2013, and the ESPEN micronutrient guideline, the micronutrient content was calculated by multiplying the value provided on the nutrition label for each product by the daily energy dose of 1500 or 1800 Kcal/day (Iacone et al., 2016). The micronutrient content was evaluated as the average content of all enteral formulas labeled as such, by formula type group (standard, semi‐elemental, or disease‐specific formulas), registration type group (ENPs or FSMPs), and by single product.

## 3 RESULTS AND DISCUSSION

Enteral formulas are usually designed according to the DRIs and provide vital substrates in the form of macronutrients, micronutrients, and antioxidants, which are essential in satisfying the daily needs of all patients, ranging from the hypermetabolic critically ill to the stable home enteral population (Doley, 2022; Limketkai et al., 2019). However, as has been said above, the micronutrients in oversupply or insufficient supply in enteral formulas could be harmful, particularly in patients on long‐term total enteral nutrition.

**FIGURE 1:**
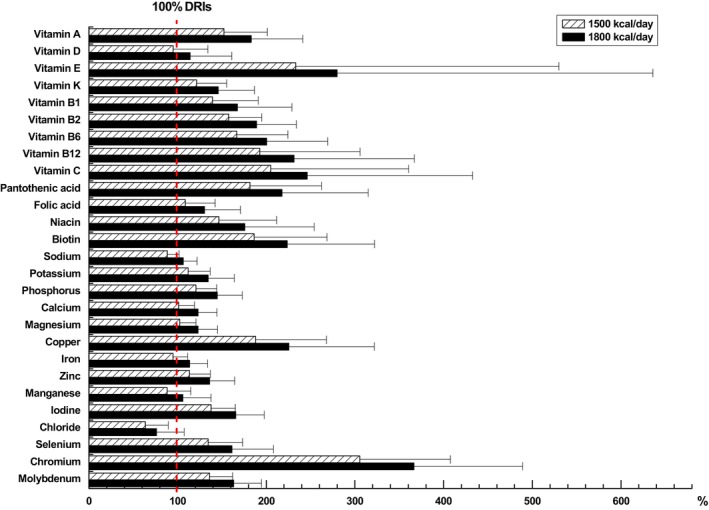
Mean percent of micronutrient content compared with the dietary reference intakes (DRIs). Bars (medium for 1500 Kcal/day and black for 1800 Kcal/day) indicate the mean calculated content of micronutrients in the 31 enteral formulas, compared to the DRIs (fixed to 100% as vertical dotted line).

**FIGURE 2:**
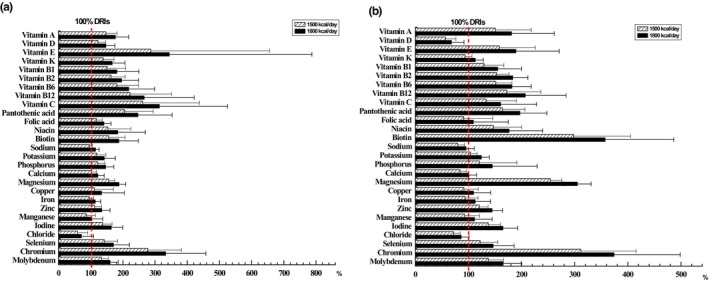
Mean percent of micronutrient content compared with the dietary reference intakes (DRIs). Bars (medium for 1500 Kcal/day and black for 1800 Kcal/day) indicate the mean calculated content of micronutrients in the enteral formulas (a: foods for special medical purposes, b: enteral nutrition preparations) compared to the DRIs (fixed to 100% as a vertical dotted line).

Tables 1 and 2 summarized the DRIs, UL, the limits established by GB 29922‐2013, the ESPEN micronutrient guideline, and the average micronutrient content of the products grouped as formula type groups at the daily energy doses of 1500 and 1800 Kcal/day, respectively. Setting the DRIs as a 100% baseline, Figure 1 showed the mean percent of calculated micronutrient content in all enteral formulas compared with the DRIs. The statistics indicated that at the daily energy doses of 1500 Kcal/day, the average content of mineral elements (sodium and chlorine), vitamin D, and trace elements (iron, manganese) did not reach the DRIs in all groups, whereas other micronutrients were covered by the DRIs. At the daily energy doses of 1800 Kcal/day, only the average content of chlorine did not reach the DRIs. When the daily energy doses were 1500 or 1800 Kcal/day, the average content of all the micronutrients met the limit requirements of GB 29922‐2013 and did not exceed the UL, regardless of the group of enteral formulas considered. Table 3 summarized the DRIs, UL, the limits established by GB 29922‐2013, the ESPEN micronutrient guideline, and the average micronutrient content of the products grouped as registration type groups at the daily energy doses of 1500 Kcal/day, respectively. Figure 2a,b indicated the average percent of calculated micronutrient content in different registration type formulas compared with the DRIs. The data showed that at the daily energy doses of 1500 Kcal/day, the average content in sodium, chlorine, iron, and manganese of FSMP formulas and the average content in vitamin D, vitamin K, folic acid, sodium, calcium, magnesium, iron, manganese, and chlorine of ENP formulas were not covered by the DRIs. At the daily energy doses of 1800 Kcal/day, the average content in vitamin D, chlorine, and sodium of ENP formulas did not reach the DRIs, and only chlorine in FSMP formulas did not reach the DRIs. When the daily energy doses were 1500 or 1800 Kcal/day, the average content of all the micronutrients met the limit requirements of GB 29922‐2013 and did not exceed the UL, except vitamin D in ENP formulas.

**TABLE 1:** DRIs for men and women (m/f), daily tolerable upper limits (UL), ESPEN micronutrient guideline, GB 29922‐2013 and micronutrient content (mean and range) in standard, semi‐elemental, and disease‐specific enteral formulas (calculated as daily intake of 1500 Kcal/day).

Micronutrient	DRIs m/f	UL	ESPEN guideline min–max (high requirements)^a^	GB 29922 limit min–max	Standard formulas *N* = 20 mean (min–max)^b^	Semi‐elemental formulas *N* = 4 mean (min–max)^b^	Disease‐specific formulas *N* = 7 mean (min–max)^b^
Vitamin A.ug RE	820/700	3000	900–1500 (1500)	585.0–3375.0	1181.3 ± 325.7 (697.3–1742.9)	1316.1 ± 229.1 (1092.0–1631.8)	1457.5 ± 732.1 (697.3–2670.4)
Vitamin D.ug	15	50	25 (30)	12.00–47.10	16.6 ± 5.6 (4.4–27.6)	13.8 ± 3.0 (10.7–16.9)	9.1 ± 4.5 (4.4–16.9)
Vitamin E.mg	14	700	15 (40)	>12.00	36.0 ± 50.2 (10.0–246.6)	24.0 ± 3.6 (20.7–28.9)	30.9 ± 22.1 (10.0–75.3)
Vitamin K.ug	80	/	120 (120)	>66.00	105.0 ± 31.0 (52.7–157.5)	94.1 ± 13.1 (79.7–108.6)	79.3 ± 6.7 (66.5–87.9)
Vitamin B_1_.mg	1.6/1.3	/	1.5 (100)	>1.05	2.3 ± 1.0 (1.3–5.0)	2.4 ± 0.3 (1.9–2.5)	2.0 ± 0.6 (1.3–2.5)
Vitamin B_2_.mg	1.5/1.2	/	1.2 (10)	>1.05	2.4 ± 0.6 (1.9–4.4)	2.5 ± 0.5 (1.9–3.1)	2.2 ± 0.3 (1.9–2.5)
Vitamin B_6_.mg	1.6	60	1.5 (7.5)	>1.05	2.8 ± 1.1 (1.9–6.3)	3.0 ± 0.6 (2.5–3.8)	2.3 ± 0.5 (1.9–3.1)
Vitamin B_12_.ug	2.4	/	2.5 (7.5)	>1.95	4.9 ± 3.2 (1.9–15.1)	4.2 ± 1.1 (3.1–5.6)	4.3 ± 1.9 (2.5–7.5)
Vitamin C.mg	100	2000	100 (200)	>84	204.7 ± 83.0 (59.6–414.2)	176.2 ± 30.2 (150.0–219.7)	242.3 ± 309.0 (59.6–930.7)
Pantothenic acid.mg	5	/	5 (7.5)	>4.35	9.3 ± 4.4 (4.4–21.3)	10.2 ± 2.1 (8.2–12.6)	8.3 ± 4.0 (4.4–15.7)
Folic acid.ug	400	1000	330–400 (500)	>333.0	441.3 ± 113.0 (129.3–627.6)	632.2 ± 378.6 (433.0–1200.0)	345.1 ± 203.7 (129.3–623.2)
Niacin.mg	16/13	310	18 (40)	>3.00	21.4 ± 9.8 (8.8–52.1)	21.7 ± 8.9 (11.9–30.1)	18.3 ± 7.9 (8.2–27.0)
Biotin.ug	40	/	30 (75)	>33.0	64.2 ± 25.2 (37.7–129.3)	63.1 ± 5.7 (56.5–69.0)	100.1 ± 46.7 (59.6–166.3)
Sodium.mg	1600	/	/	>1245	1449.0 ± 208.4 (996.0–1895.4)	1540.1 ± 202.9 (1334.3–1820.0)	1296.4 ± 245.3 (919.4–1533.9)
Potassium.mg	2200	/	/	>1665	2270.9 ± 566.0 (1494.3–4016.6)	2443.6 ± 432.5 (2001.4–3012.5)	2083.6 ± 204.1 (1776.7–2330.9)
Phosphorus.mg	720	3500	/	>600.0	847.5 ± 159.9 (527.8–1077.0)	1026.6 ± 165.0 (886.2–1236.4)	858.1 ± 204.5 (574.9–1080.1)
Calcium.mg	1200	2000	/	>840	1170.6 ± 233.9 (667.1–1569.0)	1241.9 ± 155.5 (1068.8–1443.5)	980.6 ± 225.1 (574.9–1200.0)
Magnesium.mg	330	/	/	>274.5	343.2 ± 62.5 (229.1–520.9)	367.8 ± 85.2 (278.7–483.3)	312.8 ± 40.3 (252.9–359.6)
Copper.ug	800	8000	1000–3000	660–7500	1375.6 ± 598.1 (815.9–2691.8)	1962.8 ± 685.9 (1318.0–2699.9)	1872.5 ± 597.0 (1294.7–2699.9)
Iron.mg	16/20	42	18–30 (30)	12.45–34.50	18.5 ± 3.0 (13.2–23.8)	22.4 ± 2.1 (20.1–24.5)	18.6 ± 4.1 (13.2–23.8)
Zinc.mg	12.5/9	40	10–20 (20)	6.0–33.0	13.4 ± 2.6 (8.2–18.8)	17.3 ± 1.5 (15.7–18.8)	15.0 ± 3.5 (10.0–18.2)
Manganese.ug	4500	11,000	2000–3000	375–9165	3834.8 ± 1282.3 (859.8–6056.3)	4740.7 ± 797.4 (3621.3–5509.1)	4069.6 ± 1026.8 (2689.9–5192.8)
Iodine.ug	120	600	150–300	>100.5	157.4 ± 30.3 (119.2–219.7)	203.5 ± 32.3 (179.5–251.0)	169.2 ± 24.3 (132.4–200.2)
Chloride.mg	2500	/	/	<3270	1345.4 ± 663.3 (251.0–2510.4)	1445.5 ± 519.5 (759.4–1875.3)	1536.5 ± 269.2 (1062.5–1854.6)
Selenium.ug	60	400	50–150 (200)	49.5–333.0	82.0 ± 25.6 (49.6–156.3)	90.7 ± 4.8 (85.4–95.4)	74.7 ± 20.2 (49.6–112.3)
Chromium.ug	35	/	35–150 (200)	27.0–834.0	82.8 ± 25.6 (50.2–125.5)	106.1 ± 8.0 (100.4–111.7)	98.7 ± 38.2 (66.5–180.1)
Molybdenum.ug	100	900	50–250 (250)	84.0–750.0	130.3 ± 26.5 (99.8–163.2)	166.3 ± 23.1 (150.0–182.6)	136.3 ± 23.6 (99.8–166.3)

Abbreviations: DRIs, dietary reference intakes; ESPEN, European Society for Clinical Nutrition and Metabolism.

^a^ Increased requirements during critical illness and in patients with acute admission with malnutrition (NRS ≥ 5).

^b^ The range of micronutrient content (min–max) were calculated by multiplying the value provided on the nutrition label for each standard, semi‐elemental, and disease‐specific enteral formula by the daily energy dose of 1500 Kcal/day, respectively.

**TABLE 2:** DRIs for men and women (m/f), daily tolerable upper limits (UL), ESPEN micronutrient guideline, GB 29922‐2013, and micronutrient content (mean and range) in standard, semi‐elemental, and disease‐specific enteral formulas (calculated as daily intake of 1800 Kcal/day).

Micronutrient	DRIs m/f	UL	ESPEN guideline min–max (high requirements)^a^	GB 29922 limit min–max	Standard formulas *N* = 20 mean (min–max)^b^	Semi‐elemental formulas *N* = 4 mean (min–max)^b^	Disease‐specific formulas *N* = 7 mean (min–max)^b^
Vitamin A.ug RE	820/700	3000	900–1500 (1500)	702–4050.0	1417.6 ± 390.8 (836.7–2091.4)	1579.3 ± 274.9 (1310.4–1958.1)	1749.0 ± 878.5 (836.7–3204.5)
Vitamin D.ug	15	50	25 (30)	14.40–56.52	19.9 ± 6.7 (5.3–33.1)	16.6 ± 3.6 (12.8–20.3)	11.0 ± 5.4 (5.3–20.3)
Vitamin E.mg	14	700	15 (40)	>14.40	43.2 ± 60.2 (12.0–296.0)	28.8 ± 4.3 (24.9–34.6)	37.1 ± 26.5 (12.0–90.4)
Vitamin K.ug	80	/	120 (120)	>79.20	126.0 ± 37.2 (63.3–189.0)	113.0 ± 15.7 (95.6–130.3)	95.2 ± 8.1 (79.8–105.4)
Vitamin B_1_.mg	1.6/1.3	/	1.5 (100)	>1.26	2.8 ± 1.1 (1.5–6.0)	2.8 ± 0.4 (2.3–3.0)	2.4 ± 0.7 (1.5–3.0)
Vitamin B_2_.mg	1.5/1.2	/	1.2 (10)	>1.26	2.9 ± 0.8 (2.3–5.3)	3.0 ± 0.6 (2.3–3.8)	2.7 ± 0.4 (2.3–3.0)
Vitamin B_6_.mg	1.6	60	1.5 (7.5)	>1.26	3.3 ± 1.3 (2.3–7.5)	3.6 ± 0.7 (3.0–4.5)	2.8 ± 0.6 (2.3–3.8)
Vitamin B_12_.ug	2.4	/	2.5 (7.5)	>2.34	5.9 ± 3.8 (2.3–18.1)	5.1 ± 1.3 (3.8–6.8)	5.2 ± 2.3 (3.0–9.0)
Vitamin C.mg	100	2000	100 (200)	>100.8	245.7 ± 99.6 (71.5–497.1)	211.4 ± 36.2 (180.0–263.6)	290.8 ± 370.8 (71.5–1116.9)
Pantothenic acid.mg	5	/	5 (7.5)	>5.22	11.2 ± 5.3 (5.3–25.6)	12.2 ± 2.5 (9.8–15.1)	10.0 ± 4.8 (5.3–18.8)
Folic acid.ug	400	1000	330–400 (500)	>399.6	529.5 ± 135.6 (155.1–753.1)	758.6 ± 454.4 (519.7–1440.0)	414.1 ± 244.4 (155.1–747.8)
Niacin.mg	16/13	310	18 (40)	>3.60	25.7 ± 11.8 (10.5–62.5)	26.0 ± 10.7 (14.3–36.1)	21.9 ± 9.4 (9.8–32.4)
Biotin.ug	40	/	30 (75)	>39.6	77.0 ± 30.2 (45.2–155.1)	75.7 ± 6.9 (67.8–82.8)	120.1 ± 56.1 (71.5–199.6)
Sodium.mg	1600	/	/	>1494	1738.8 ± 250.1 (1195.2–2274.4)	1848.2 ± 243.5 (1601.1–2184.0)	1555.7 ± 294.4 (1103.3–1840.6)
Potassium.mg	2200	/	/	>1998	2725.0 ± 679.2 (1793.2–4820.0)	2932.3 ± 519.0 (2401.7–3615.0)	2500.4 ± 244.9 (2132.1–2797.1)
Phosphorus.mg	720	3500	/	>720	1017.0 ± 191.9 (633.4–1292.4)	1231.9 ± 198.0 (1063.4–1483.6)	1029.7 ± 245.5 (689.9–1296.1)
Calcium.mg	1200	2000	/	>1008	1404.7 ± 280.7 (800.6–1882.8)	1490.2 ± 186.6 (1282.6–1732.2)	1176.7 ± 270.1 (689.9–1440.0)
Magnesium.mg	330	/	/	>329.4	411.9 ± 74.9 (274.9–625.1)	441.3 ± 102.3 (334.4–579.9)	375.4 ± 48.3 (303.5–431.5)
Copper.ug	800	8000	1000–3000	792–9000	1650.7 ± 717.8 (979.1–3230.1)	2355.4 ± 823.1 (1581.6–3239.9)	2247.0 ± 716.4 (1553.7–3239.9)
Iron.mg	16/20	42	18–30 (30)	14.94–41.40	22.2 ± 3.6 (15.8–28.6)	26.9 ± 2.5 (24.1–29.4)	22.4 ± 5.0 (15.8–28.6)
Zinc.mg	12.5/9	40	10–20 (20)	7.2–39.6	16.1 ± 3.1 (9.8–22.6)	20.7 ± 1.8 (18.8–22.6)	18.0 ± 4.2 (12.0–21.8)
Manganese.ug	4500	11,000	2000–3000	450–10,998	4601.8 ± 1538.8 (1031.8–7267.6)	5688.9 ± 956.9 (4345.5–6610.9)	4883.6 ± 1232.2 (3227.9–6231.3)
Iodine.ug	120	600	150–300	>120.6	188.9 ± 36.4 (143.1–263.6)	244.2 ± 38.8 (215.4–301.2)	203.0 ± 29.1 (158.9–240.2)
Chloride.mg	2500	/	/	<3924	1614.5 ± 795.9 (301.2–3012.5)	1734.6 ± 623.4 (911.3–2250.3)	1843.9 ± 323.0 (1275.0–2225.5)
Selenium.ug	60	400	50–150 (200)	59.4–399.6	98.4 ± 30.7 (59.5–187.5)	108.8 ± 5.8 (102.4–114.5)	89.6 ± 24.3 (59.5–134.8)
Chromium.ug	35	/	35–150 (200)	32.4–1000.8	99.4 ± 30.7 (60.2–150.6)	127.3 ± 9.6 (120.5–134.1)	118.5 ± 45.8 (79.8–216.1)
Molybdenum.ug	100	900	50–250 (250)	100.8–900.0	156.4 ± 31.8 (119.7–195.8)	199.6 ± 27.7 (180.0–219.2)	163.5 ± 28.3 (119.7–199.6)

Abbreviations: DRIs, dietary reference intakes; ESPEN, European Society for Clinical Nutrition and Metabolism.

^a^ Increased requirements during critical illness and in patients with acute admission with malnutrition (NRS ≥ 5).

^b^ The range of micronutrient content (min–max) was calculated by multiplying the value provided on the nutrition label for each standard, semi‐elemental, and disease‐specific enteral formula by the daily energy dose of 1800 Kcal/day, respectively.

**TABLE 3:** DRIs for men and women (m/f), daily tolerable upper limits (UL), ESPEN micronutrient guideline, GB 29922‐2013, and micronutrient content (mean and range) in foods for special medical purpose and enteral nutrition preparations (calculated as daily intake of 1500 Kcal/day).

Micronutrient	DRIs m/f	UL	ESPEN guideline min–max (high requirements)^a^	GB 29922 limit min–max	FSMPs (*N* = 19) mean (min–max)^b^	ENPs (*N* = 12) mean (min–max)^b^
Vitamin A.ug RE	820/700	3000	900–1500 (1500)	585–3375	1205.2 ± 283.9 (746.8–1631.8)	1312.3 ± 550.5 (697.3–2670.4)
Vitamin D.ug	15	50	25 (30)	12–47.1	18.4 ± 3.5 (15.1–27.6)	8.2 ± 3.0 (4.4–11.9)
Vitamin E.mg	14	700	15 (40)	>12	40.1 ± 51.7 (15.1–246.6)	22.7 ± 9.5 (10.0–37.7)
Vitamin K.ug	80	/	120 (120)	>66	111.2 ± 26.4 (79.7–157.5)	75.2 ± 10.1 (52.7–87.2)
Vitamin B_1_.mg	1.6/1.3	/	1.5 (100)	>1.05	2.4 ± 0.9 (1.3–5.0)	2.0 ± 0.6 (1.3–2.5)
Vitamin B_2_.mg	1.5/1.2	/	1.2 (10)	>1.05	2.4 ± 0.7 (1.9–4.4)	2.3 ± 0.4 (1.9–2.8)
Vitamin B_6_.mg	1.6	60	1.5 (7.5)	>1.95	2.9 ± 1.1 (1.9–6.3)	2.4 ± 0.5 (1.9–3.2)
Vitamin B_12_.ug	2.4	/	2.5 (7.5)	>1.05	5.3 ± 3.1 (2.5–15.1)	4.1 ± 1.5 (1.9–10.0)
Vitamin C.mg	100	2000	100 (200)	>84	261.0 ± 176.5 (113.0–930.7)	130.6 ± 52.9 (59.6–225.9)
Pantothenic acid.mg	5	/	5 (7.5)	>4.35	10.3 ± 4.4 (5.0–21.3)	8.0 ± 2.8 (4.4–16.3)
Folic acid.ug	400	1000	330–400 (500)	>333	469.0 ± 74.1 (375.3–627.6)	405.1 ± 169.9 (129.3–1200.0)
Niacin.mg	16/13	310	18 (40)	>3	21.5 ± 10.0 (8.2–52.1)	20.1 ± 7.9 (8.8–33.3)
Biotin.ug	40	/	30 (75)	>33	62.4 ± 20.1 (37.7–100.4)	121.2 ± 43.0 (39.5–497.7)
Sodium.mg	1600	/	/	>1245	1528.2 ± 152.5 (1318.0–1895.4)	1249.8 ± 204.5 (919.4–1510.6)
Potassium.mg	2200	/	/	>1265	2366.6 ± 573.3 (1882.8–4016.6)	2064.5 ± 253.6 (1494.3–2330.9)
Phosphorus.mg	720	3500	/	>600	876.8 ± 154.4 (633.9–1236.4)	847.8 ± 199.6 (527.8–1080.1)
Calcium.mg	1200	2000	/	>840	1222.0 ± 193.8 (941.4–1569.0)	977.5 ± 200.5 (574.9–1288.8)
Magnesium.mg	330	/	/	>274.5	366.1 ± 57.4 (295.0–520.9)	297.7 ± 40.4 (229.1–345.2)
Copper.ug	800	8000	1000–3000	660–7500	1250.9 ± 475.5 (815.9–2372.3)	1994.5 ± 563.4 (1294.7–2699.9)
Iron.mg	16/20	42	18–30 (30)	12.45–34.5	19.0 ± 2.8 (15.1–24.5)	18.5 ± 4.3 (13.2–23.8)
Zinc.mg	12.5/9	40	10–20 (20)	6–33	13.9 ± 2.7 (8.2–18.8)	14.8 ± 3.4 (10.0–18.2)
Manganese.ug	4500	11,000	2000–3000	375–9165	3878.9 ± 1263.2 (859.8–6056.3)	4101.3 ± 1087.8 (2689.9–5509.1)
Iodine.ug	120	600	150–300	>100.5	163.8 ± 35.6 (119.2–251.0)	164.0 ± 27.6 (113.0–200.2)
Chloride.mg	2500	/	/	<3270	1301.9 ± 689.2 (251.0–2510.4)	1571.7 ± 274.4 (1062.5–2024.6)
Selenium.ug	60	400	50–150 (200)	49.5–333	85.2 ± 24.7 (50.2–156.3)	73.5 ± 19.8 (49.6–112.3)
Chromium.ug	35	/	35–150 (200)	27–834	83.2 ± 31.3 (50.2–125.5)	92.2 ± 31.1 (66.5–180.1)
Molybdenum.ug	100	900	50–250 (250)	84–750	133.2 ± 21.4 (113.0–163.2)	136.0 ± 28.3 (99.8–182.6)

Abbreviations: DRIs, dietary reference intakes; ESPEN, European Society for Clinical Nutrition and Metabolism.

^a^ Increased requirements during critical illness and in patients with acute admission with malnutrition (NRS ≥ 5).

^b^ The range of micronutrient content (min–max) was calculated by multiplying the value provided on the nutrition label for each FSMP and ENP by the daily energy dose of 1500 Kcal/day, respectively.

The present results confirmed that most of the micronutrients provided by enteral formulas were usually above the DRIs for a healthy crowd, below the UL, within the range of the relevant Chinese standards and the ESPEN micronutrient guideline, at a calorie intake of 1500 or 1800 Kcal/day. However, at the calorie intake of normal diets, the sodium and chlorine content in the products are often intentionally kept lower than the DRIs. The main reason may be that the risk of disease caused by a high‐salt diet ranks first among all the unhealthy dietary factors in China. For healthy people or patients, appropriate limits on sodium intake are good for their health (Chinese Nutrition Society, 2013). And as far as we know, there are no reports researching the possible adverse effects of inadequate chlorine intake in enteral formulas for patients on long‐term enteral nutrition; meanwhile, the daily intake of chlorine may also come from other sources, such as city water (Chinese Nutrition Society, 2013). Moreover, the sodium and chlorine content of enteral formulas also keep within the limits set by GB 29922‐2013. Therefore, we believe that the sodium and chlorine content of the products evaluated do not need much attention.

**FIGURE 3:**
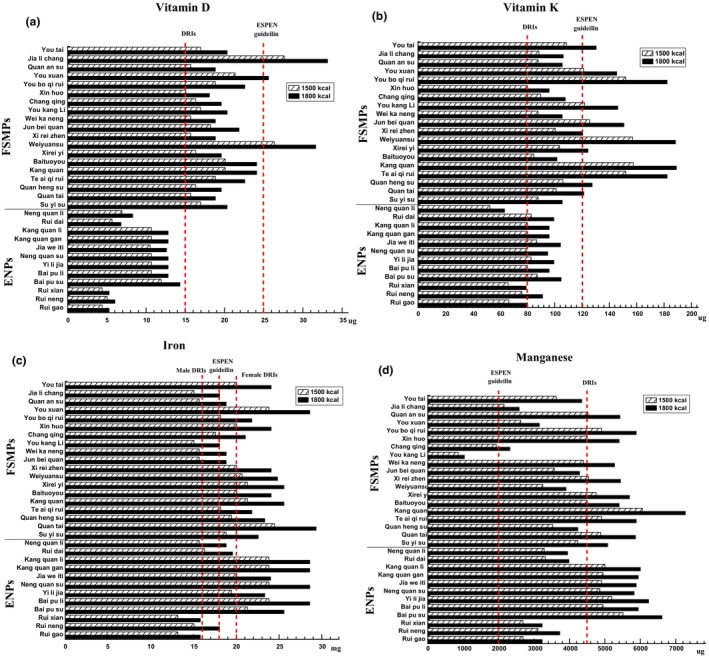
Certain micronutrients content in the enteral formulas evaluated. For each enteral formula evaluated, the calculated content of vitamin D (a), vitamin K (b), iron (c), and manganese (d) at doses of 1500 or 1800 Kcal/day (as medium bars for 1500 Kcal/day and black bars for 1800 Kcal/day). The value of the dietary reference intakes (DRIs) and the European Society for Clinical Nutrition and Metabolism (ESPEN) micronutrient guideline at 1500 and 1800 Kcal/day (as a vertical dotted line).

The average content of vitamin D in all assessed enteral formulas at the calorie intake of 1500 Kcal/day was about 7% lower than recommended by the DRIs, which currently provided an average content of about 14 μg/day (Figure 1). Figure 3a showed that when the vitamin D content of 31 enteral formulas was compared by single product, in 39% of the formulas studied, the intake of the common daily dose of 1500 Kcal was insufficient to cover the established DRIs, and the formulas all belong to ENPs. Patients requiring nutritional therapy will frequently be depleted/deficient in vitamin D because of low intake and a lack of ultraviolet light: Their need may therefore be significantly higher (Berger et al., 2022; Cámara‐Martos & Iturbide‐Casas, 2019). There are many large and relevant risk groups for vitamin D deficiency, including patients with severe kidney or liver dysfunction, bedridden, and chronically ill patients (Cashman et al., 2016). The recommendation of the ESPEN clinical nutrition guideline is an authoritative opinion in the field of enteral nutrition and has an important guiding role in how to carry out enteral nutrition support. The guideline suggests that enteral nutrition should provide at least 1000 IU (25 μg) per day of vitamin D in 1500 Kcal because patients on EN frequently receive 400–800 IU (10–20 μg)/day (Berger et al., 2022). Although this may be adequate in some patients, the above dose is higher because patients receiving EN are likely to have higher requirements because of poor status resulting from prior illness. However, currently commercial enteral formulas contain less than the minimum recommended 1000 IU (25 μg) for most patients in stable clinical conditions and without relevant metabolic diseases, and even more so the 1200 IU (30 μg) for patients with critical illness and acute admission with malnutrition (nutritional risk screening; NRS ≥ 5) (Cámara‐Martos & Iturbide‐Casas, 2019). Therefore, we suggest that the vitamin D content in the enteral formulas be increased to the limit recommended by the ESPEN micronutrient guideline as well as within the limit established by GB 29922‐2013.

The data of the present study showed that the average content of vitamin K provided was about 95 μg at a calorie intake of 1500 Kcal/day in these enteral formulas, which was comparable with the DRIs for a healthy population (Figure 1), but it was lower than as recommended by the ESPEN micronutrient guideline. The guideline suggests that enteral nutrition in adults should provide at least 120 μg of vitamin K per day with 1500 Kcal (Berger et al., 2022). At a calorie intake of 1500 Kcal/day, by comparing the micronutrient content of a single formula, the results from Figure 3b indicated that vitamin K content in 16% of the formulas (5 ENPs) studied was insufficient to cover the established DRIs, and 23% of the formulas (7 FSMPs) was above the recommended levels of the ESPEN micronutrient guideline. This may be due to the fact that the food source of vitamin K is abundant and the vitamin K can be synthesized by the intestinal microflora of normal humans, so vitamin K deficiency rarely occurs in normal people (Chinese Nutrition Society, 2013). However, patients who suffer from fat malnutrition, malabsorption (cystic fibrosis, celiac disease, short bowel, etc.), and antibiotic and anticoagulant (warfarin) treatments are most likely to suffer from vitamin K deficiency (Berger et al., 2022). So when the above patients require long‐term enteral nutritional support, the intake of vitamin K should be taken into consideration. Supplements can be prescribed if needed. In addition, for patients with anticoagulant treatments, it is important to consider the significant impact of EN on anticoagulation response in patients on vitamin K antagonists and an adjustment in the drug administration with a 1‐h interruption of enteral nutrition before and after anticoagulant administration (Heldt & Loss, 2013). For these reasons, we advise that the vitamin K content in the enteral formulas be appropriately increased to the limit recommended by the ESPEN micronutrient guideline.

The average content of iron in the studied enteral formulas at the calorie intake of 1500 Kcal/day was about 19 mg per day, which is comparable with the recommendation of the DRIs (Figure 1) and close to the minimum of the ESPEN micronutrient guideline. The guideline suggests that enteral nutrition in adults should provide 18–30 mg of iron per day with 1500 Kcal (Berger et al., 2022). Figure 3c showed that at a dose of 1500 Kcal, iron content in a single formula compared to the DRIs and the ESPEN micronutrient guideline. With regard to iron content, 29% of the formulas (4 ENPs and 5 FSMPs) researched are less than the recommended value of the DRIs for males, and 52% of the formulas (6 ENPs and 10 FSMPs) are not enough to cover the established DRIs for females. As well, 35% of the formulas (5 ENPs and 6 FSMPs) are below the recommended levels of the ESPEN micronutrient guideline. Iron deficiency is generally common in disease populations. The greatest risk of deficiency is observed in patients with chronic diseases due to frequent blood sampling (e.g., dialysis patients), patients after major surgery, or patients after bariatric surgery (Berger et al., 2022). Additionally, when dealing with hospitalized patients with an inflammatory bowel disease, iron deficiency is the most common extraintestinal symptom and may be prevalent in as many as 74% of the patients (Forbes et al., 2017). Therefore, we suggest that for patients on long‐term enteral nutrition support, the modestly higher doses of iron provided in the estimated enteral formulas are likely to be beneficial and not harmful, considering the high prevalence of iron deficiency.

The manganese average content supplied about 3960 μg at a calorie intake of 1500 Kcal/day in the evaluated enteral formulas was about 13% lower than recommended by the DRIs (Figure 1). The ESPEN micronutrient guideline suggests that enteral nutrition in adults should provide 2000–3000 μg manganese per day, but doses up to 6000 μg/day have been safely provided in 1500 Kcal (Berger et al., 2022). Although the average content supplied by products was almost twofold more than the recommended lower limit of the ESPEN guideline, it was still within the limits set by GB 29922‐2013. At a calorie intake of 1500 Kcal/day, comparing the manganese content of single formulas, Figure 3d indicated that the manganese content in 48% of the products (5 ENPs and 10 FSMPs) studied was insufficient to cover the established DRIs, and the content in all products was more than the recommended minimum levels of the ESPEN micronutrient guideline except for two FSMP formulas. Few studies on manganese deficiency due to long‐term enteral nutritional support are reported. In critically ill patients, the proportion of patients with decreased values was low (2.1%) compared with other trace elements such as zinc or copper; in addition, high or low values do not seem to be related to outcome (Lee et al., 2019). Based on the above reasons, in our opinion, the manganese content in all evaluated enteral formulas does not represent a concern.

There are several possible reasons for the difference in micronutrient content between different enteral formulas and the recommendations of the DRIs. Firstly, because of the different formula types for enteral formulas and the wide range of daily energy intake for individuals with different diseases, the daily intake of micronutrients is related to the dose administered. If the patients are in stable clinical conditions and without relevant metabolic diseases, the requirements for micronutrients seem to be very close to the recommendations of the DRIs for the general population. Standard formulas are often used for those patients in clinical practice, so the micronutrient content in standard formulas should be as close as possible to the DRIs. For patients with special diseases, the requirements for micronutrients are significantly different from those of the normal population. Therefore, a few micronutrients in disease‐specific formulas are adjusted according to specific needs or clinical conditions compared with standard formulas. For example, potassium, phosphorus, and magnesium concentrations are lower in renal formulas to decrease the buildup of these renally excreted electrolytes in patients with kidney injury; chromium‐enriched formulas for diabetics to improve glycemic control; and formulas enriched with micronutrients that have antioxidant properties (vitamin E, zinc, copper, and selenium) for patients affected by burns and wound healing. Secondly, compared with FSMP formulas, more micronutrients in ENP formulas deviate from the DRIs. This is mainly because when ENPs were introduced into China in the 1970s and 1980s and regulated as pharmaceuticals, the Chinese DRIs had not been established at that time. Although the first edition of the Chinese DRIs was published in 2000, it is difficult to renew the ENP formula based on the DRIs due to pharmaceuticals. However, all micronutrients in FSMP formulas keep within the limits set by GB 29922‐2013, whose minimum and maximum limit values are developed according to the DRIs, and the minimum limit is close to the DRIs. Therefore, FSMP formulas are more suitable for patients on long‐term enteral nutrition support than ENP formulas. Furthermore, in order to meet the needs of the DRIs and the labels stated, most manufacturers add amounts of micronutrients above the recommended value of the DRIs and the levels specified on the labels to compensate for the potential losses of these micronutrients during production and storage. Therefore, the true value of some micronutrients in these formulas may be far higher than the recommended value of the DRIs or the labeled value. If the patients accept long‐term total enteral nutrition, it is without doubt a matter of concern.

## 4 CONCLUSION

In a word, most enteral formulas on the Chinese market are generally suitable for patients on long‐term total enteral nutrition, and FSMP formulas are more suitable than ENP formulas. We suggest that the vitamin D, vitamin K, and iron content in these enteral formulas be appropriately increased to the limit recommended by the ESPEN micronutrient guideline. The results could provide a basis for research and development of more suitable enteral formulas by manufacturers and help to administer more effective enteral nutrition support for patients on long‐term total enteral nutrition by clinical dietitians in clinical practice, especially individualized treatment.

## AUTHOR CONTRIBUTIONS

**Hong Yang:** Conceptualization (equal); data curation (equal); formal analysis (equal); funding acquisition (equal); investigation (equal); methodology (equal); writing – original draft (equal); writing – review and editing (equal). **Ling Hou:** Data curation (equal); formal analysis (equal); investigation (equal); methodology (equal); writing – original draft (equal). **Hong Mei Sun:** Data curation (equal); investigation (equal); methodology (equal); writing – original draft (equal). **Shu Hong Ye:** Conceptualization (equal); funding acquisition (equal); project administration (equal); supervision (equal); writing – review and editing (equal).

## CONFLICT OF INTEREST STATEMENT

The authors declare that they have no conflict of interest.

## ETHICS STATEMENT

This study does not contain any human or animal testing.

## Data Availability

The data that validate the results of this research are available from the corresponding author upon reasonable request.
